# Reply to: Drug-drug interactions between palbociclib and proton pump inhibitors may significantly affect clinical outcome of metastatic breast cancer patients

**DOI:** 10.1016/j.esmoop.2022.100393

**Published:** 2022-02-04

**Authors:** R. Beechinor, A. Palumbo, H.K. Chew, M. Arora

**Affiliations:** 1 UC Davis Comprehensive Cancer Center, Sacramento, USA; 2 University of California, San Francisco School of Pharmacy, San Francisco, USA; 3 Department of Pharmacy, Oregon Health and Science University, Portland, USA; 4 Division of Hematology and Oncology, University of California Davis Comprehensive Cancer Center, University of California Davis School of Medicine, Sacramento, USA

Del Re et al. report that concomitant use of proton pump inhibitors (PPIs) has a detrimental effect on progression-free survival (PFS) in patients with metastatic breast cancer (mBC) treated with palbociclib.^1^ The authors describe a retrospective observational analysis of mBC patients treated with palbociclib, and they report that those who take PPIs for at least two-thirds of their treatment have a PFS of 14.0 months, compared to 37.9 months (*P* < 0.0001) in patients not taking PPIs.^1^ There are several critical considerations omitted in this analysis that call into question these findings and the resulting conclusions presented.

Firstly, the authors have failed to recognize and account for the negative impact PPIs have on overall mortality. This has been demonstrated in both meta-analyses of all patient types and in patients with cancer, specifically breast cancer where the hazard ratio for increased risk for mortality has been estimated at 1.43 (95% confidence interval 1.24-1.47).^2^, ^3^, ^4^, ^5^ This effect likely biases the comparison carried out by Del Re et al., and may obfuscate the etiology of this PFS difference as a drug-interaction concern, when it may in fact be driven by PPI use independent of drug interactions. Secondly, the magnitude of reduction in PFS demonstrated is not likely biologically plausible given the purported mechanism suggested for this interaction. The authors cite literature suggesting that PPI-induced high gastric pH leads to a reduction in systemic plasma concentrations of palbociclib and thus decreased efficacy.^1^ However, the difference in median PFS they report between subjects receiving PPIs and those who did not (37.9 versus 14.0 months, Δ = 23 months) is numerically greater than the difference in PFS reported between palbociclib and placebo in the PALOMA-2 (24.8 versus 14.5 months, Δ = 10.3 months) and PALOMA-3 trials (11.2 versus 4.6 months, Δ = 6.6 months).^1^^,^^6^^,^^7^ This would not be explained by a reduction in systemic palbociclib exposure alone, where one would expect subjects receiving PPIs to experience similar or greater efficacy than placebo subjects. Rather, this may suggest that PPI use is somehow correlated with reduced PFS by an altogether different mechanism. Importantly, preclinical data suggest that PPIs may have anti-proliferative effects against breast tumor cells, reinforcing the suggestion that detrimental effects of PPIs on patient outcomes are not likely related to anticancer effects via drug interactions.^8^ A third concern is that the authors describe this as retrospective research; however, they do not detail how they identified the exact number of subjects in each comparison group (*n* = 56 subjects received PPIs versus *n* = 56 subjects did not). This raises concerns regarding potential selection bias for subjects who were evaluated in this comparison. Lastly, nowhere in their manuscript do the authors specify the formulation of palbociclib used in their study. This is critically important as Pfizer recently re-formulated palbociclib from a capsule into a tablet, which no longer needs to be taken with food, and relevant studies with concomitant PPI use have not demonstrated its impact on absorption.^9^^,^^10^

We agree with the authors that PPIs are often over-prescribed, and stewardship is needed to avoid long-term exposure in patients without appropriate indications for therapy. However, we feel it is crucial that the readership is aware of these concerns we have raised, which preclude any definitive conclusions regarding this potential interaction, as these may contribute toward misconceptions regarding the efficacy of palbociclib, or lead to inappropriate avoidance of PPI use when clinically indicated.
