# Erratum to ‘Reply to: Drug-drug interactions between palbociclib and proton pump inhibitors may significantly affect clinical outcome of metastatic breast cancer patients’ [ESMO Open Volume 7, Issue 1, February 2022, 100393]

**DOI:** 10.1016/j.esmoop.2022.100460

**Published:** 2022-03-17

**Authors:** R. Beechinor, A. Palumbo, H.K. Chew, M. Arora

**Affiliations:** 1 UC Davis Comprehensive Cancer Center, Sacramento; 2 University of California, San Francisco School of Pharmacy, San Francisco; 3 Department of Pharmacy, Oregon Health and Science University, Portland; 4 Division of Hematology and Oncology, University of California Davis Comprehensive Cancer Center, University of California Davis School of Medicine, Sacramento, USA

The publisher regrets that the affiliations for the authors in this article were incorrect at the time the article was published. They are correct as presented within this erratum.

The publisher would like to apologise for any inconvenience caused.

